# A comparative study on the efficacies of gonadotropin-releasing hormone (GnRH) agonist and GnRH antagonist in neoadjuvant androgen deprivation therapy combined with transperineal prostate brachytherapy for localized prostate cancer

**DOI:** 10.1186/s12885-016-2737-8

**Published:** 2016-09-01

**Authors:** Kenta Miki, Hiroshi Sasaki, Masahito Kido, Hiroyuki Takahashi, Manabu Aoki, Shin Egawa

**Affiliations:** 1 Department of Urology, Jikei University School of Medicine, 3-25-8 Nishi-Shimbashi, Minato-ku, 105-8461 Tokyo, Japan; 2 Department of Pathology, Jikei University School of Medicine, Tokyo, Japan; 3 Department of Radiology, Jikei University School of Medicine, Tokyo, Japan

**Keywords:** Prostate cancer, Brachytherapy, GnRH antagonist, GnRH agonist, Neoadjuvant androgen deprivation therapy, Prostate specific antigen, Quality of life, Testosterone

## Abstract

**Background:**

Neoadjuvant androgen deprivation therapy (ADT) has been suggested to confer several clinical benefits in patients with prostate cancer (PCa) undergoing transperineal prostate brachytherapy (TPPB). Unlike gonadotropin-releasing hormone (GnRH) receptor agonists, a GnRH antagonist such as degarelix can achieve castrate levels of testosterone without testosterone flare. However, normalization of serum testosterone levels following completion of neoadjuvant ADT in either form of treatment has never been compared in clinical trials.

**Methods/Design:**

This is a single-center, open-label, randomized controlled study that will compare the efficacy and safety of degarelix with those of existing GnRH agonists combined with ^125^I-TPPB. A total of 56 patients with low/intermediate-risk clinically localized PCa will be enrolled and randomized to one of two treatment groups: the GnRH agonist group and the degarelix group. Patients in the GnRH agonist group will receive leuprorelin acetate or goserelin acetate, and those in the degarelix group will receive the initial dose of 240 mg as 2 subcutaneous injections of 120 mg each, and then 80 mg of maintenance doses every 4 weeks for 12 weeks. Those randomly assigned to the 12-week intervention period will subsequently undergo 48-weeks of follow-up after ^125^I-TPPB. The primary endpoint is defined as normalization of serum testosterone levels (>50 ng/dL) following completion of neoadjuvant ADT. All patients will be assessed every 4 weeks for the first 24 weeks, then every 12 weeks for the next 36 weeks after administrations of these drugs. Secondary endpoints are the proportion of normalized serum luteinizing hormone (LH) and follicle-stimulating hormone (FSH), the percent reduction in prostate specific antigen (PSA) compared with pretreatment levels, the percent reduction in total prostate volume (TPV) during neoadjuvant ADT, the percent increase in TPV after ^125^I-TPPB, the percent reduction in hemoglobin, serum alkaline phosphatase (ALP), changes in free testosterone and bone mineral content measurement, the proportion of patients who have serum testosterone levels over 50 ng/dL at 12 weeks following completion of neoadjuvant ADT, and the improvement of quality of life (QOL).

**Discussion:**

The present study will provide additional insight regarding the benefit and potency of degarelix and will examine its potential as a new option for administration in neoadjuvant ADT.

**Trial registration:**

Identification number: UMIN000015519.

Registration date: October 24, 2014.

## Background

Androgen deprivation therapy (ADT) that effectively reduces serum testosterone levels has been a core tool for treating metastatic and advanced prostate cancer (PCa) [1]. It is also an integral part of definitive treatment in combination with radiotherapy in the management of localized and locally advanced diseases [2, 3]. In Japan, ^125^I-transperineal prostate brachytherapy (TPPB) has been approved as one of the definitive options to treat localized PCa since 2003 [4]. Efficacy of neoadjuvant and adjuvant ADT using gonadotropin-releasing hormone (GnRH) agonists and anti-androgen with ^125^I-TPPB are currently tested in a phase III, multicenter, randomized, controlled trial (Seed and Hormone for Intermediate-risk Prostate Cancer (SHIP) 0804 study) [5].

Some studies have shown that patients treated with neoadjuvant ADT have fewer positive surgical margins but without improving biochemical control after radical prostatectomy [6, 7]. A significant reduction in total prostate volume (TPV) after 3 to 8-month neoadjuvant ADT has been reported [8–12]. Although GnRH agonists have been used for many years as ADT, they may be associated with a counterintuitive initial testosterone surge that can delay castration and which may stimulate PCa cells, resulting in potentially detrimental exacerbation of clinical symptoms particularly in advanced diseases [13]. An alternative approach to ADT has emerged in the form of a GnRH antagonist that involves the direct and rapid blockade of GnRH receptors, producing rapid suppression of testosterone and prostate specific antigen (PSA) levels. The effect occurs more rapidly than with GnRH agonists, without testosterone flare. Studies that evaluate the optimal agents and duration of ADT that produce outcomes with fewer adverse events are thus important.

Treatment with ADT is not avoid of adverse events, such as fatigue, diminished sexual function hot flushes and most importantly cardiovascular disease(CVD) which mainly due to a suppression of testosterone [14–16]. Many studies have shown testosterone recoveries after discontinuance of ADT. The extent and time to normalization of serum testosterone are relevant to the pre-treatment patients’ characteristics such as ages, treatment duration, pretreatment testosterone level, species, Gleason score and the level of dihydroxytestosterone [17–21]. However, most of those studies are retrospectively designed and inconclusive owing to the unavailability of pretreatment testosterone. Regarding adverse events, the results from previous studies are controversial and confusing. Shore ND et al. stated the potential advantages of GnRH antagonists in adverse events and oncological outcome [22], while Kimura T et al. questioned the real advantage of that drug [23]. In this study, we hypothesized 3 months GnRH antagonist to be more advantageous than GnRH agonists owing to more rapid recovery of serum testosterone after discontinuation. This may result in reduced incidence of ADT-related adverse events.

We describe our study protocol for low/intermediate-risk PCa, which is a single-center, open-label, randomized controlled study of a 12-week intervention period as neoadjuvant ADT followed by 48-weeks follow-up after ^125^I-TPPB. Japanese regulations specify the maximum permitted number of seeds for use, and the maximum intensity of radiation [5, 24]. To comply with these requirements, it is our common practice to administer neoadjuvant ADT even for low- to intermediate-risk PCa in patients with relatively large prostate glands (≥40 ml). In this study, we will evaluate temporal changes in serum testosterone levels and TPV before and after the discontinuation of short-term degarelix and GnRH agonist administration. The final goal of this study is to establish an appropriate strategy in neoadjuvant ADT for PCa without testosterone surge or microsurges by using short-term degarelix administration combined with ^125^I-TPPB.

## Methods/Design

### Aim of the study

To perform a comparative study between GnRH antagonist, degarelix and GnRH agonists on the recovery of serum testosterone levels for low/intermediate risk PCa after neoadjuvant ADT combined with ^125^I-TPPB. GnRH antagonist is hypothesized to have significantly more rapid testosterone recovery after discontinuation.

### Study design

**Fig. 1:**
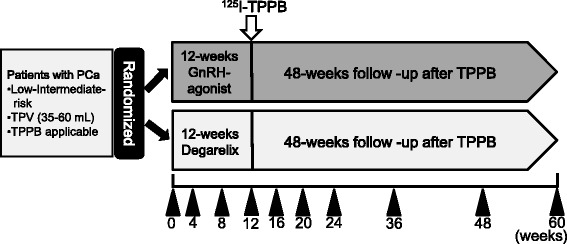
Study design (UMIN000015519). Patients who meet the inclusion criteria are enrolled and randomized. Patients receive 12 weeks’ neoadjuvant therapy with GnRH agonists or degarelix followed by 48 weeks of follow-up after ^125^I-TPPB. Arrowheads show the time points for assessments

The present study is designed as a single-center, open-label, randomized controlled study to be performed in patients with low/intermediate-risk PCa. The outline of the study protocol is shown in Fig. 1. All patients are randomized to one of two treatment groups in which patients receive 12 weeks neoadjuvant therapy with either GnRH agonists or antagonist followed by 48 weeks of follow-up after ^125^I-TPPB.

### Intervention

All eligible patients will be assigned randomly to one of two groups, the GnRH antagonist group and the GnRH agonist group. The initial dose of degarelix is 240 mg given as 2 subcutaneous injections of 120 mg each at 40 mg/ml in the abdomen. After the initial dose, the maintenance dose of 80 mg is given as one subcutaneous injection in the abdomen at 20 mg/ml, every 4 weeks. Leuprorelin acetate is administered subcutaneously once every 4 weeks at a dose of 3.75 mg, and goserelin acetate is administered subcutaneously in the abdomen once every 4 weeks at a dose of 3.6 mg.

### Informed consent-ethics approval

This study was conducted in accordance with the Helsinki Declaration of 1975, as revised in 2000. All treatments for PCa are undertaken following written informed consent. Study approval was granted by the Jikei University Ethics Committee Institutional Review Board (approval No. 25–366 ((7501)), date June 2, 2014).

### Technique of ^125^I-TPPB

^125^I-TPPB for all patients is administered using an ultrasound-guided technique with either the Mick applicator or intraoperatively built custom linked seed technique [5, 24, 25]. The implant is planned to deliver a dose of at least 144 Gy to the clinical target volume, which includes the prostatic gland and treatment margin [26]. Although individual technical aspects are institution-dependent, efforts are made to assure optimal quality control of the radiation dose based on our over 1,000 cases of experience [27]. Computed tomography images, taken at 2–5 mm intervals, are obtained 1 month after ^125^I-TPPB to determine the extent of edema. Dose-volume histograms for the prostate, urethra, and rectum are computed to obtain post-planning distribution data. V100 and D90 should be over 95 % and 144 Gy respectively for the clinically targeted volume [26, 28].

### Definition of endpoints

#### Primary endpoints

The primary endpoint is defined as normalization of serum testosterone (>50 ng/dL) after discontinuation of GnRH agonists and antagonists.

#### Secondary endpoints

Secondary endpoints are: 1) the proportion of normalized serum luteinizing hormone (LH) and follicle-stimulating hormone (FSH), 2) the percent reduction in PSA compared with pretreatment levels, 3) the percent reduction in TPV during neoadjuvant ADT, 4) the percent increase in TPV after ^125^I-TPPB, 5) the percent reduction in hemoglobin and serum alkaline phosphatase, 6) changes in free testosterone and bone mineral content measurement, 7) the proportion of patients who have serum testosterone levels over 50 ng/dL at 12 weeks after completion of neoadjuvant ADT, 8) the improvement of QOL using the international prostate symptom score (IPSS) for lower urinary tract symptoms, 9) the improvement of QOL using the Expanded Prostate Cancer index Composite (EPIC), and 10) the improvement of QOL using the international index of erectile function (IIEF5). The assessment schedule is shown as Table 1.

**Table 1:**
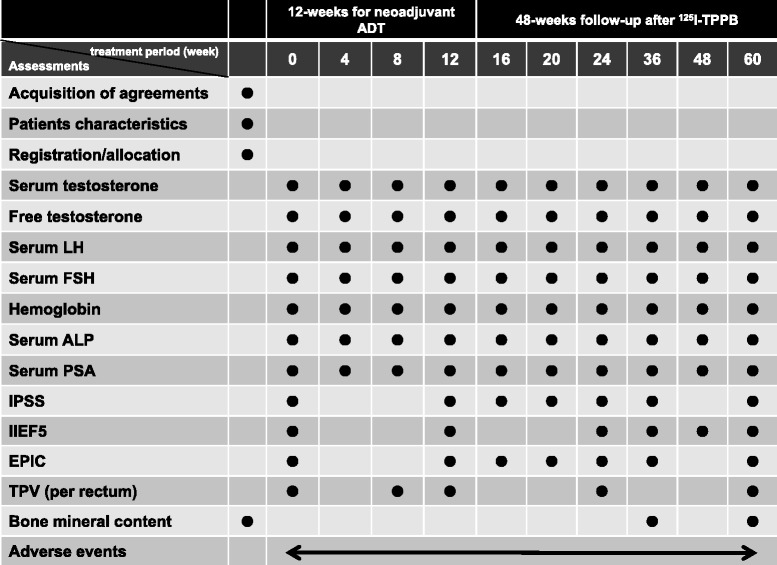
Assessment schedule

Closed circles indicate when each assessment is performed. Patients are monitored for unwanted symptoms and adverse events throughout the study period

### Eligibility criteria-inclusion criteria

Patients must:

- Be at least 20 years of age, with a definitive histological diagnosis of PCa by needle biopsy.
- Be adaptable to ^125^I-TPPB.
- Have low/intermediate-risk localized PCa as follows: low-risk PCa: cT1a-T2a, Gleason score 2–6 and PSA < 10 ng/ml; intermediate-risk PCa: cT2b-T2c or Gleason score 7 or PSA 10–20 ng/ml (excluding Gleason score ≥ 8, PSA ≥ 20 ng/ml).
- Have TPV 35–60 ml.
- Agree in writing to participate in this clinical study after receiving adequate explanation.

### Eligibility criteria-exclusion criteria

Patients are ineligible if they:

- Have previously received hormonal therapies including GnRH agonists, GnRH antagonists, antiandrogen agents, estrogen agents or orchiectomy for PCa.
- Are using 5α reductase inhibitors.
- Have severe asthma (e.g. use inhaled corticosteroid that is necessary for daily life), anaphylactic reaction, severe urticaria and complication or medical history of angioedema.
- Are sensitive to mannitol.
- Have multiple malignancies.
- Have alanine aminotransferase (ALT) ≥ 100 IU/L or total bilirubin ≥ 1.3 mg/dL.
- Are for any other reason considered by the investigator to be inappropriate for participation in the present study.

### Tracking and monitoring adverse events

Patients will be monitored for unwanted symptoms and adverse events throughout the study period. Adverse events reported spontaneously by the patient or observed by physicians are similarly assessed and recorded. They all must be reported to the principal investigator and will be followed until they have abated or until a stable situation has been reached.

### Data collection

This study design was chosen to ensure accurate, standardized, and high-quality data collection. All patients giving written informed consent to the study are asked to complete a short family history and epidemiology questionnaire. Electronic Data Capture (EDC) systems licensed by Pharma Consulting Group are used to collect clinical data in electronic format, with clinical data being obtained from patient medical records. A follow-up data form is completed by the investigator at week 4, 8, 12, 24, 36 and 48 after ^125^I-TPPB. These forms capture information regarding patient characteristics, serum testosterone, free testosterone, LH, FSH, serum ALP, hemoglobin, PSA, IPSS, IIEF5, EPIC, TPV, bone mineral content and adverse events.

### Statistical consideration

#### Sample size

This study is designed to examine the superiority of GnRH antagonist over agonists in terms of testosterone recovery after its discontinuation. In the previous studies that investigated the time to normalization of testosterone levels after discontinuation of GnRH agonist administration, 50 % of patients with clinically localized PCa showed more than castration level testosterone (>50 ng/dL) within 4–5 months after 3-month GnRH agonist treatment [29, 30]. By contrast, the time to normalization of testosterone levels for degarelix treatment was 1.6 [31] or 2 months [32]. The hazard ratio calculated from both of median survival for time to normalization of testosterone levels was approximately 3.1. Assuming the hazard ratio of 3.1, it was found that a sample size of 26 patients per group would be necessary using the log-rank test with a significance level of 5 and power of 80 %. Assuming that 5 % dropout rate, the target sample was set at 28 patients per group (56 patients in total).

#### Statistical methods

Statistical analyses will be performed on an intention-to-treat basis. Serum testosterone levels after neoadjuvant ADT will be tested and normalization of serum testosterone levels above castration level (50 ng/dL) will be defined as event. Survival curves will be estimated using the Kaplan-Meier method. The log-rank test will be used to test the differences between the two groups. The hazard ratio will be estimated using the Cox proportional hazard model. The longitudinal change of QOL scores (IPSS, IIEF5 and EPIC) following ^125^I-TPPB will also be compared between groups. Patients will be evaluated for toxicity, graded according to the National Cancer Institute Common Toxicity Criteria version 4.0 (https://ctep.cancer.gov/protocolDevelopment/electronic_applications/ctc.htm#ctc_40). All tests will be two-sided, and a *p*-value of 0.05 will be considered statistically significant.

#### Methods of recruitment and randomization

Recruiting began in 2015. Eligible patients are randomly assigned to one of two treatment groups through the EDC system. Randomization is done centrally using dynamic allocation [33] to obtain good between-group balance for factors including age category (<68/ ≥ 68) and the TPV (<45 mL/ ≥ 45 mL) before administrations of degarelix and GnRH agonists. The probability to be assigned to the group of lowest imbalance is set to 0.8.

#### Patient enrollment and anticipated completion of enrollment

Our current expectation is that the final patient will be enrolled by March, 2017; the study will be clinically completed by April, 2018 and results will be available during the third quarter of 2018.

## Discussion

Some previous studies that have investigated the impact of ADT on intermediate- to high-risk PCa treated with ^125^I-TPPB suggested clinical advantages for the addition of ADT to ^125^I-TPPB [34, 35]. Lee et al. [34] reported that hormonal therapy consisting of LH-releasing hormone agonist combined with an antiandrogen for 3 months before brachytherapy and continued for 2–3 months afterward significantly improved 5-year actuarial freedom from biochemical failure, 79 % vs 54 % without hormonal therapy. Contrary to these reports, there are several reports showing that neoadjuvant ADT did not improve outcome for any risk group [36], and a large retrospective matched-pair analysis failed to show benefit of neoadjuvant ADT combined with either ^125^I-TPPB or ^103^Pd-TPPB [37]. Thus, there is still controversy regarding the impact of ADT on intermediate to high-risk PCa treated with ^125^I-TPPB and the most effective and safe treatment strategy remains to be established. It should be critical for designing the study protocol to take into consideration of the agents, the duration and the optimal timing of ADT combined with ^125^I-TPPB. Additionally, potential adverse events such as fatigue, diminished sexual function, and hot flushes, caused by this treatment should be taken into consideration. Although the optimal duration of concomitant ADT for intermediate-risk PCa when combined with ^125^I-TPPB remains unknown until the results from SHIP0804 study [5] are available, it may be possible to minimize the duration of ADT and its related toxicities for patients who achieve a rapid fall in testosterone and PSA after starting neoadjuvant ADT. Shortening this intervention period will be expected to reduce costs and side effects, and to improve QOL [38].

The agents that are mainly used as adjuvant ADT include estrogens, anti-androgen monotherapy, and combined androgen blockade using an anti-androgen plus a GnRH receptor agonist [1]. However, despite their efficacy, GnRH agonists have several drawbacks associated with their mechanism of action, including an initial testosterone surge. Compared to GnRH agonist, degarelix, a recently approved GnRH receptor antagonist, can achieve castration levels of testosterone much faster, without the risks associated with testosterone flare. Mason et al. recently conducted a comparative study for the use of degarelix and GnRH agonist in neoadjuvant ADT in combination with radiotherapy, and reported that a short-period such as 12-weeks of degarelix treatment achieved comparable efficacy with that of goserelin plus bicalutamide as neoadjuvant ADT before radiotherapy [39].

Note that our study protocol also focuses on the evaluation of temporal change of testosterone levels and TPV downsizing after withdrawal of degarelix, comparing it with that of existing GnRH agonists, in neoadjuvant ADT combined with ^125^I-TPPB. Although testosterone suppression is the primary outcome and it has been used as a surrogate endpoint during the approval of several hormonal treatments, only a few studies evaluated serum testosterone levels after the discontinuations of GnRH agonists [29, 30] and degarelix administrations [31]. Given that the TPV downsized with GnRH agonists, it might be expected that serum testosterone would be restored to normal levels immediately. However, suppression of testosterone levels remained and continued to lower even more than a half year after the discontinuation of GnRH agonist administrations [19, 40]. By contrast, the normalization of testosterone level to more than castration level after discontinuation of degarelix treatment was 1.6 [31] or 2 months [32]. Therefore, it is interesting to conduct a comparative study on the efficacy and safety for degarelix and GnRH agonists after the simultaneous discontinuation of these treatments, however, no such study has reported so far.

In conclusion, the present study is conducted to prospectively evaluate the efficacy and safety of degarelix, comparing it with the existing GnRH agonist, in neoadjuvant ADT for patients with low/intermediate-risk PCa. We expect that degarelix will prove to be an effective and well-tolerated agent, providing a useful addition to the hormonal armamentarium for PCa, and offering patients with hormone-sensitive disease a valuable alternative treatment option in neoadjuvant ADT.

## Abbreviations

ADT: Androgen deprivation therapy
EBRT: External beam radiotherapy
EDC: Electronic data capture
EPIC: Expanded prostate cancer index composite
FSH: Follicle-stimulating hormone
GnRH: Gonadotropin-releasing hormone
IIEF5: International index of erectile function
IPSS: International prostate symptom score
LH: Luteinizing hormone
PCa: Prostate cancer
PSA: Prostate specific antigen
QOL: Quality of life
TPPB: Transperineal prostate brachytherapy
TPV: Total prostate volume
